# *Centella asiatica* enhances diabetic wound healing by decreasing macrophage-driven inflammation *via* the AKT/MAPK/NF-κB pathway

**DOI:** 10.3389/fphar.2025.1632573

**Published:** 2025-09-29

**Authors:** Fugang Xiao, Qin Li, Wenwen Zeng, Botao Tang, Qingqing Chen, Chengjie Wu, Zixiao Duan, Huan Chen, Shunli Rui, Bin Liu

**Affiliations:** ^1^ Department of Endocrinology and Metabolism, Chongqing University Central Hospital, Chongqing Emergency Medical Centre, School of Medicine, Chongqing University, Chongqing, China; ^2^ Department of Endocrinology, the First Affiliated Hospital of Chongqing Medical University, Chongqing, China; ^3^ Department of Geriatrics, Bazhong Central Hospital, Bazhong, China; ^4^ Department of Emergency, Chongqing University Central Hospital, Chongqing Emergency Medical Center, Chongqing, China

**Keywords:** DFU, *Centella asiatica*, pharmacology, inflammation, diabetic wound healing

## Abstract

**Background:**

Diabetic foot ulcers (DFU) pose a major global healthcare challenge, necessitating novel therapeutic strategies. Although *Centella asiatica* has demonstrated pharmacological potential, its role in diabetic wound healing remains unclear. This study aims to investigate the mechanisms through which *Centella asiatica* promotes wound healing under diabetic conditions.

**Methods:**

A systematic pharmacological approach was employed to identify potential targets of *Centella asiatica* in DFU. *In vitro* experiments used Raw264.7 macrophages stimulated by advanced glycation end products (AGEs). For *in vivo* validation, a diabetic mouse model was established using high-fat diet and streptozotocin (STZ). Full-thickness skin wounds were topically treated with *Centella asiatica* at concentrations of 200 μg/cm^2^ and 300 μg/cm^2^.

**Results:**

*Centella asiatica* significantly suppressed AGE-induced nitric oxide (NO) overproduction and downregulated inducible nitric oxide synthase (iNOS) and pro-inflammatory cytokines in Raw264.7 cells. It also promoted anti-inflammatory factor expression. Mechanistically, it activated the AKT/MAPK/NF-κB pathway. In diabetic mice, *Centella asiatica* accelerated wound closure, with the most effective dose observed at 200 μg/cm^2^.

**Conclusion:**

This study demonstrates that *Centella asiatica* facilitates diabetic wound healing through anti-inflammatory mechanisms involving the AKT/MAPK/NF-κB pathway. These findings provide mechanistic support for the potential integration of *Centella asiatica* into DFU management.

## Introduction

Diabetic foot ulcer (DFU) represents a formidable complication of diabetes, exhibiting a 22% amputation rate and placing considerable strain on patients, their families, and the healthcare system (Armstrong et al., 2017; Wang et al., 2021; Zhang et al., 2020). The persistence of such wounds is often attributed to factors such as peripheral vascular disease, neuropathy, poor glycemic control, and a heightened infection risk (Armstrong et al., 2023). Standard clinical interventions for DFU include surgical debridement, decompression, management of lower limb ischemia, and infection control (Margolis et al., 2011). Nonetheless, these treatments frequently fall short in facilitating swift and thorough healing. As comprehension of disease mechanisms grows, herbal remedies are increasingly being considered as adjuvant or alternative approaches for various conditions. The development of treatments capable of regulating blood sugar and enhancing wound healing simultaneously could offer an effective strategy for diabetic wound management.

Naturopathic approaches, noted for their therapeutic potency and safety profile, are gaining traction in disease prevention and treatment, frequently offering fewer side effects compared to synthetic medications (Caesar et al., 2021; Wu et al., 2024; Zou et al., 2022). *Centella asiatica*, a plant with a history in traditional medicine, has shown promise in the management of diabetic complications, as evidenced by recent findings on its anti-inflammatory, immunomodulatory, and antioxidant capabilities (Bindal et al., 2024; Bylka et al., 2014). Its pronounced anti-inflammatory properties have garnered considerable interest and have been linked to enhanced wound healing (Chi et al., 2021; Hashim et al., 2011; Liu et al., 2024; Lokanathan et al., 2016; Wang et al., 2024). Importantly, bacterial infections, often consequent to prolonged inflammation, are a principal factor in the delayed healing of diabetic wounds; nevertheless, the specific interactions between *Centella asiatica* and diabetic wound repair have yet to be fully elucidated (Fan et al., 2021). Although some studies have confirmed that Centella asiaticacan promote chronic wound healing by regulating related pathways, they only focus on the *in vitro* mechanism of a single component and do not involve the interaction between advanced glycation end product accumulation and inflammatory networks in the diabetic microenvironment; other some studies have investigated the healing effect of *Centella asiatica* extract on diabetic rat wounds, but have not deeply analyzed the synergistic effects of its active components and downstream molecular targets. We emphasize that while previous studies focused on single compounds (e.g., asiaticoside) or general anti-inflammatory effects, our study integrates systematic pharmacology with multi-level validation to explore the synergistic effects of multiple metabolites within the diabetic microenvironment. Building on these premises, the objective of this study is to investigate the therapeutic potential of *Centella asiatica* in the management of diabetic trauma and to elucidate its underlying mechanisms of action.

In this study, we employed a systematic pharmacological approach, complemented by both *in vivo* and *in vitro* validation methods, to thoroughly assess the therapeutic potential of *Centella asiatica* in the treatment of diabetic wounds. Integrating findings from existing literature and pharmacological screening (Alqahtani et al., 2015), we identified Cynarine, Quercetin, and Kaempferol as the principal active constituents of *Centella asiatica*, which were characterized using high-performance liquid chromatography (HPLC). Through a systematic pharmacological enrichment analysis, we discovered that these compounds are significantly involved in the regulation of various factors associated with inflammation, including members of the matrix metalloproteinase (MMP) family, as well as estrogen receptor 1 (ESR1) and estrogen receptor 2 (ESR2). Further *in vitro* investigations demonstrated that *Centella asiatica* effectively reduces AGEs-related inflammation in Raw264.7 cells, potentially through the inhibition of the iNOS/NO and AKT/MAPK/NF-κB signaling pathways. This modulation appears to influence the expression of a diverse array of inflammatory mediators, encompassing pro-inflammatory cytokines such as IL-1β, IL-6, and TNF-α, as well as anti-inflammatory cytokines like IL-4 and IL-10. Moreover, the therapeutic efficacy of these interventions was corroborated by subsequent *in vivo* evaluations. These findings contribute to a partial understanding of the mechanisms through which *Centella asiatica* may promote wound healing, thereby holding substantial implications for the progression of both fundamental research and clinical applications of Traditional Chinese Medicine (TCM) in the treatment of DFU.

## Materials and methods

### Network construction

Utilizing Traditional Chinese Medicine Systems Pharmacology (TCMSP, https://www.tcmsp-e.com), Swiss Target Prediction (http://www.swisstargetprediction.ch), and the PubChem database (https://pubchem.ncbi.nlm.nih.gov), we identified protein targets associated with *Centella asiatica*. Additionally, we extracted DFU-related target proteins from the human genome database GeneCards (https://www.genecards.org) and the UniProt database (https://www.uniprot.org). Through Venn diagram analysis, we identified overlapping targets between DFU and *Centella asiatica*, which were subsequently considered potential therapeutic targets for the treatment of DFU using *Centella asiatica*.

### GO and reactome pathways enrichment analysis

GO and Reactome pathways enrichment analyses were performed to elucidate the core mechanisms and pathways underlying the anti-DFU properties of *Centella asiatica* using the STRING database (https://string-db.org) and Hiplot Website (https://hiplot.com.cn) (Li J. et al., 2022; Szklarczyk et al., 2023). A query was conducted using the STRING database to identify genes with shared targets, specifically restricting the analysis to the species “*Homo sapiens*.” This investigation encompassed GO categories, including biological processes, cellular components, and molecular functions, as well as Reactome pathways (Milacic et al., 2024).

### Cell culture and *Centella asiatica* preparation

We obtained mouse mononuclear macrophage cells (RAW 264.7) from Procell Company (Wuhan, China). These cells were cultured in RPMI 1640 medium with 11.1 mM glucose (Gibco, 12633012) at 37 °C in a humidified incubator with 5% CO2. The culture medium included 0.5% penicillin–streptomycin supplied by WelGENE Inc. (Korea), along with 10% fetal bovine serum from VivaCell (China).

*Centella asiatica* powder was supplied by Tianjiang Pharmaceutical Co., Ltd. (China). For processing, the samples should be kept at −80 °C. In addition, the active components were characterized by high-performance liquid chromatography (HPLC) analysis.

### Cell viability and nitric oxide (NO) release assay

Raw 264.7 cells were tested for viability using Apexbio’s Cell Counting Kit-8 (CCK-8, K1018), as described in the protocol. The readings were made using a Synergy™ H1 microplate reader. Cells were seeded into a 96-well plate at a density of 2 × 10^4 cells *per* well and subsequently pre-treated with 200 μg/mL AGEs for 24 h (Khan et al., 2022), followed by the intervention with centella sinensis for 24 h. No release assay was conducted using the NO fluorescence probe kit (S0019S, Beyotime Biotechnology), and subsequent procedures were carried out strictly according to the kit instructions.

### Western blot and qRT-PCR array

Proteins were extracted from cells using RIPA buffer with protease inhibitors (SC-364162, Santa Cruz, CA) and phosphatase inhibitors (B15001, Selleck). Protein concentration was measured using a BCA assay kit (ZJ102, Epizyme). Following this, the proteins were separated by polyacrylamide gel electrophoresis and transferred to a PVDF membrane (Millipore, Billerica). The membrane was blocked for 1 hour with TBST containing 5% milk, followed by overnight incubation at 4 °C with p-NF-κB (T55034, Abmart), p-AKT (T40067S, Abmart), AKT (T55561S, Abmart), p-AMPK (2531S, CST), AMPK (YT0216, ImmunoWay), iNOS (340668, Zen Bio), and β-Tubulin (WL01931, Wanleibio) antibodies. After three washes with TBST, an HRP-conjugated secondary antibody (Thermo Fisher) was applied. Antigen detection was performed using a chemiluminescence kit (NCM Biotech), visualized with a ChemiDoc Touch imaging system (Bio-Rad), and analyzed using ImageJ.

Total RNA was extracted using TRIzol™ reagent (Invitrogen) to obtain total RNA. The High-Capacity cDNA Reverse Transcription Kit (Invitrogen) was used to synthesize cDNA. iTaq™ Universal SYBR Green Supermix (Bio-Rad) was used for qRT-PCR, and the data were analyzed with CFX™96 Manager Software (Bio-Rad). To measure mRNA expression levels, we normalized them to actin.

### Immunofluorescent staining and morphological staining

After the tissues were fixed with paraformaldehyde for a duration of 16–24 h at 4 °C, 5-μm sections were subsequently prepared. The sections were subjected to deparaffinization and antigen retrieval utilizing sodium citrate, followed by a paraffin embedding step to facilitate the subsequent processing of the specimens. A blocking buffer was then applied to the sections. At 4 °C overnight incubated with antibodies specific to CD31 (R10021, Zen Bio), PCNA (27210, SAB), Cleaved-caspase3 (9661, CST), VEGF-A (WL00009b, WanLeiBio), α-SMA (55135-I-AP, Proteintech). The procedure was conducted utilizing secondary antibodies conjugated to Alexa Fluor 488 and Alexa Fluor 555 (Thermo Fisher). DAPI was used as a staining agent in the mounting medium (C1005, Beyotime). Additionally, Masson’s trichrome staining was conducted following the manufacturer’s protocol utilizing a kit (G1346, Solarbio). The quantification of collagen in the stained skin sections was performed using ImageJ software.

### T2DM induction

Male C57BL/6 J mice were obtained from Eye Biotech (Suzhou, China) and were maintained on a 12-h light/dark cycle with unrestricted access to standard chow and water. Over a period of 4 weeks, the mice were subjected to a high-fat diet (HFD, D12492; Research Diets) and received daily intraperitoneal injections of 35 mg/kg streptozotocin (STZ), which was dissolved in citrate buffer (pH 4.2–4.5), for a duration of five consecutive days. Blood samples collected from the tail were utilized to assess glucose levels. Mice exhibiting blood glucose concentrations exceeding 16.7 mmol/L after 1 week were classified as diabetic (Rui et al., 2024).

### Establishment of skin wound models and treatment

In accordance with the outlined methodology, an experimental model for wound healing was established (Xiao et al., 2024). Randomization: Male C57BL/6 J mice (n = 30) were randomly assigned to 3 groups (Centella asiatica-200 μg/cm^2^ group, Centella asiatica-300 μg/cm^2^ group, and Vehicle group) to ensure equal distribution of body weight and baseline glucose levels. Animals were anesthetized using isoflurane (RDW), and a 6 mm cylindrical biopsy tool (Health Link, Florida) was employed to create full-thickness wounds on both sides of the dorsum. Postoperatively, digital images of the wounds were captured every 2 days. *Centella asiatica* was formulated with Vaseline and administered to the wound edge at concentrations of 200ug/cm^2^ and 300ug/cm^2^, respectively, after each wound image acquisition. A standardized calibration protocol was employed to measure wound areas, and the wound closure rate was subsequently calculated using ImageJ software (National Institutes of Health, Bethesda, MD). To investigate the mechanisms of wound healing, mRNA and protein analyses were conducted on euthanized mice. Wound tissues were collected using 8 mm skin biopsy punches. The study is reported in accordance with ARRIVE guidelines (https://arriveguidelines.org).

### Statistics

In order to analyze the supplementary data, we used Prism 9.3 (GraphPad, San Diego, CA) as a statistical analysis program. Multiple groups were compared using one-way ANOVA with *post hoc* Tukey tests, while Student's t-tests were used to compare two groups. *p* < 0.05 was considered statistically significant.

## Result

### GO and reactome pathways analysis of Centella asiatica-DFU targets

**FIGURE 1:**
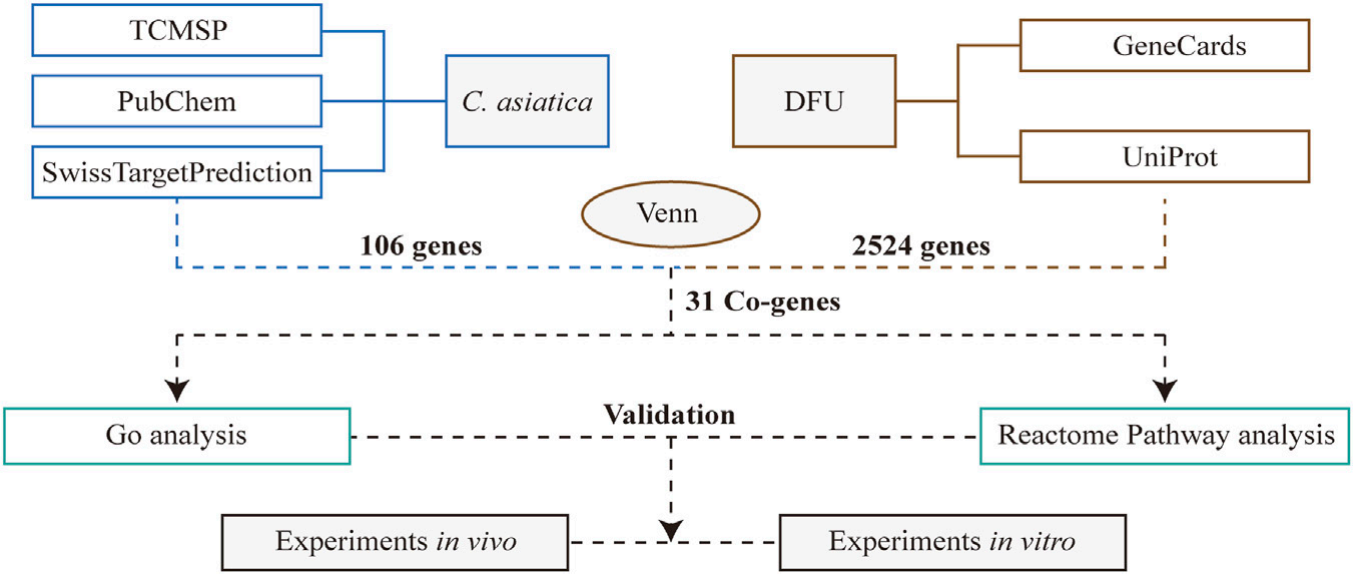
A schematic diagram of a work process.

We employed a systematic pharmacology approach to elucidate the potential mechanisms underlying the therapeutic effects of *Centella asiatica* on diabetic patients with DFU, as delineated in our workflow (Figure 1). Active phytochemicals from *Centella asiatica* were identified using the TCMSP database and extant literature, comprising three principal components: Cynarine, Quercetin, and Kaempferol. Target prediction utilized the PubChem and SwissTargetPrediction databases, yielding 106 unique targets following the removal of duplicates. From GeneCards and UniProt databases, we extracted 2,524 DFU-related targets. We constructed a Venn diagram to intersect the targets from both *Centella asiatica* and DFU, leading to the identification of 31 common targets posited as key mediators in *Centella asiatica*’s therapeutic strategy against DFU. More importantly, these prevalent targets encompass a diverse array of inflammation-associated regulators, including the matrix metalloproteinase family and estrogen receptors (Supplementary Table S1).

**FIGURE 2:**
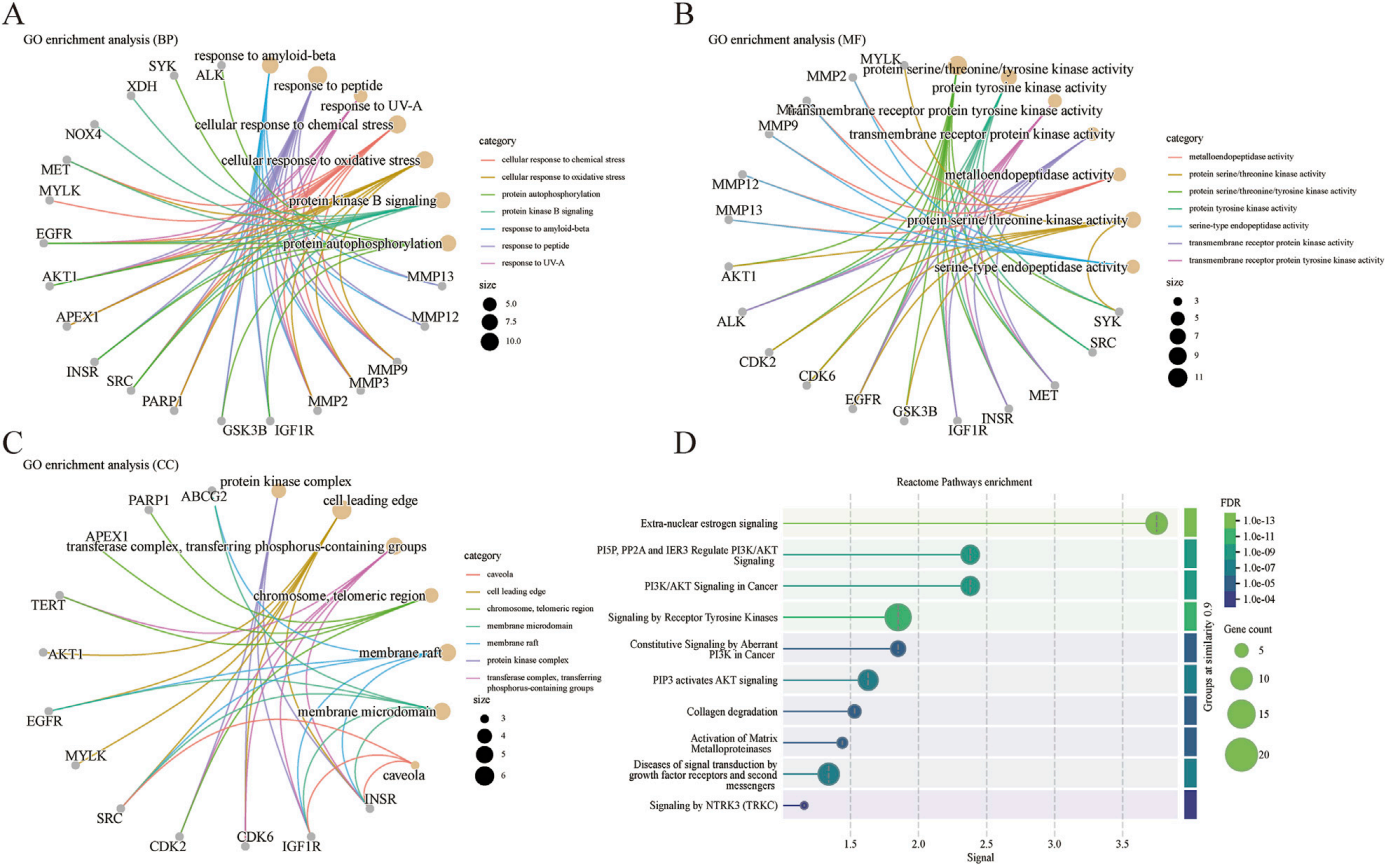
GO and Reactome pathways analysis. **(A)** Biological processes analysis. **(B)** Molecular functions analysis. **(C)** Cellular components analysis. **(D)** Reactome pathways enrichment.

To further elucidate the mechanism by which *Centella asiatica* affects DFU, we conducted a GO enrichment analysis of 31 putative *Centella asiatica* targets. The analysis identified the terms across the biological process (BP), cellular component (CC), and molecular function (MF) categories associated with *Centella asiatica*. The BP category indicated that the active compounds predominantly influence cellular response to chemical stress, response to oxidative stress, protein autophosphorylation, protein kinase B signaling, and response to amyloid-beta (Figure 2A). As for MF, the inferred targets are chiefly implicated in metalloendopeptidase activity, protein serine/threonine kinase activity, protein serine/threonine/tyrosine kinase activity, protein tyrosine kinase activity, and serine-type endopeptidase activity (Figure 2B). The CC category comprises elements such as the caveola, cell leading edge, chromosome, telomeric region, membrane microdomain, membrane raft, and protein kinase complex (Figure 2C). Collectively, these results indicate a strong association with the regulation of kinase activities, oxidative stress, and inflammatory pathways.

A subsequent Reactome pathways enrichment analysis was conducted to delineate the role of *Centella asiatica*’s functional pathways in addressing DFU targets (Figure 2D). Pathways with the highest significance included those related to extra-nuclear estrogen signaling, PI3K/AKT signaling, tyrosine kinases, collagen degradation, activation of matrix metalloproteinases, and growth factor receptors signaling in diabetic complications. In line with the GO findings, these pathways are connected to inflammatory signaling. The GO and Reactome pathways analyses enrichment together suggest that *Centella asiatica*’s mechanism chiefly constitutes anti-inflammatory effects by modulating such signals.

### *Centella asiatica* can reduce macrophage inflammation *in vitro*

**FIGURE 3:**
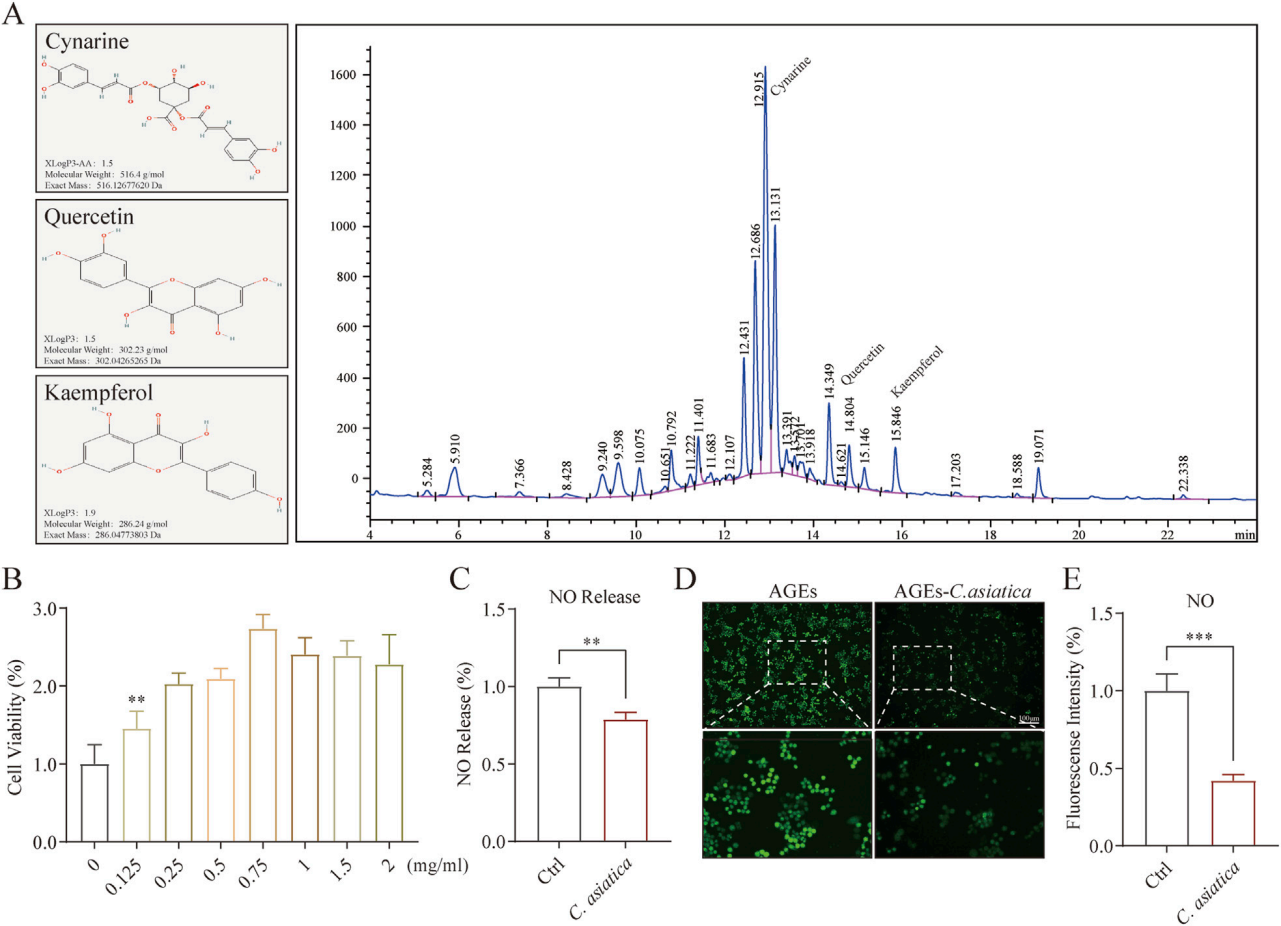
Metabolite characterization of *Centella asiatica* and ssessment of Raw264.7 function after *Centella asiatica* intervention. **(A)** The active metabolites of *Centella asiatica* were characterized by HPLC of compounds (Cynarine, Quercetin, and Kaempferol), with their molecular structures sourced from the PubChem database. **(B)** CCK-8 assay assessing cell viability of *Centella asiatica* at various concentrations 24 h post-intervention, n = 6. **(C)** NO release assay, n = 4. **(D,E)** Representative immunofluorescence images and analyses of NO (Green) release levels in Raw 264.7, n = 3 (scale bar: 100 μm). Statistical analyses were performed using GraphPad Prism 9. The results were expressed as mean ± SD. *p < 0.05, **p < 0.01, ***p < 0.001; ns, not significant.

To enhance the identification of the principal active compounds in Centella sinensis, we characterized key active substances Cynarine, Quercetin, and Kaempferol utilizing HPLC (Figure 3A). Subsequently, the anti-inflammatory effect on RAW264.7 macrophages was further studied *in vitro*. Initially, a cell viability assay established an optimal *Centella asiatica* concentration of 0.75 mg/mL for intervention (Figure 3B). Using AGEs to mimic the diabetic microenvironment *in vitro* (Xiao et al., 2024). The NO production by macrophages constitutes critical inflammatory events, which are principally driven by iNOS (Sumi and Ignarro, 2004; Yang et al., 2022). Through investigating AGEs-induced NO release, we observed a significant attenuation with *Centella asiatica* treatment (Figure 3C). Correspondingly, immunofluorescence analyses substantiated these findings (Figures 3D,E). Consequently, our preliminary findings have suggested that the intervention of *Centella asiatica* can significantly suppress macrophage-mediated inflammation, potentially serving as a critical factor in the diabetic wound healing process.

### AKT/AMPK mediates the anti-inflammatory activity of *Centella asiatica in vitro*

**FIGURE 4:**
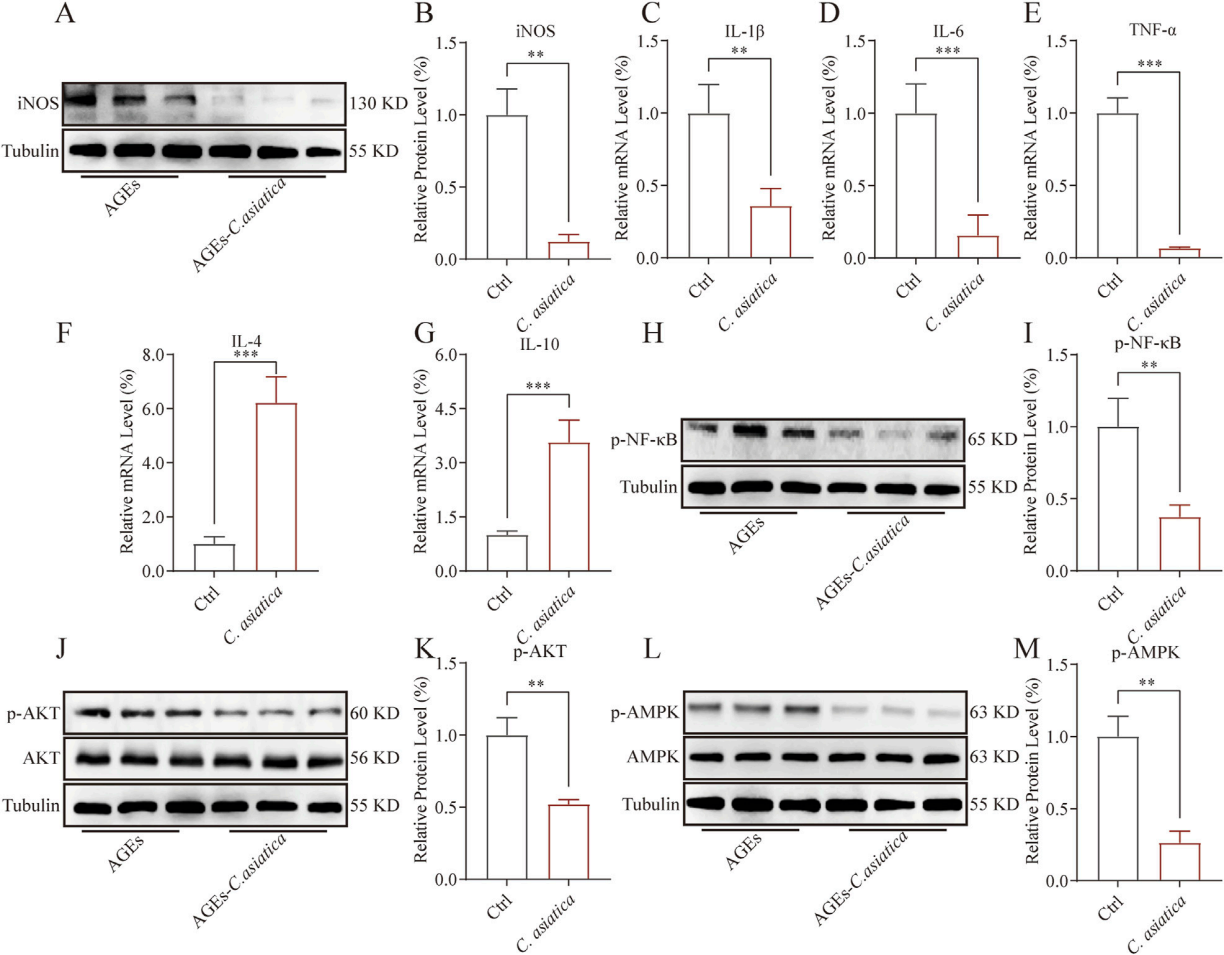
AKT/AMPK mediates the anti-inflammatory activity of *Centella asiatica in vitro*. Cells were pretreated with 200 μg/mL AGEs for 24 h, then exposed to 0.75 mg/mL *Centella asiatica* for 24 h. **(A,B)** The relative levels of iNOS in Raw 264.7 after intervention were measured by Western blot, n = 3. **(C–G)** The qRT-PCR analysis of IL-1β, IL-6, TNF-α, IL-4, and IL-10 mRNA levels following *Centella asiatica* intervention, n = 4. **(H,I)** The relative levels of p-NF-κB in Raw 264.7 after intervention were measured by Western blot, n = 3. **(J,K)** The relative levels of p-AKT in Raw 264.7 after intervention were measured by Western blot, n = 3. **(L,M)** The relative levels of p-AMPK in Raw 264.7 after intervention were measured by Western blot, n = 3. The results were expressed as mean ± SD. *p < 0.05, **p < 0.01, ***p < 0.001; ns, not significant.

To further validate the regulatory effect of *Centella asiatica* on macrophage inflammation levels, Western blot analysis was subsequently employed. The results indicated that the AGEs-induced expression of iNOS had been downregulated following intervention with *Centella asiatica* (*p* < 0.05, Figures 4A,B). Moreover, qRT-PCR analysis demonstrated that *Centella asiatica* suppressed the expression of pro-inflammatory cytokines IL-1β, IL-6, and TNF-α, markedly inhibiting their mRNA levels (*p* < 0.05, Figures 4C–E), while upregulated anti-inflammatory cytokines IL-4 and IL-10 (*p* < 0.05, Figures 4F,G). In sum, our results support the anti-inflammatory attributes of *Centella asiatica*.

NF-κB played a pivotal role in mediating the inflammatory response by initiating the transcription of inflammatory cytokines, such as TNF-α, IL-6, and IL-1β. These responses could be notably augmented by AGEs (Dong et al., 2016). In this context, we investigated the impact of *Centella asiatica* on the phosphorylation of NF-κB, confirming its efficacy in mitigating NF-κB phosphorylation (*p* < 0.05, Figures 4H,I). Furthermore, to elucidate the mechanism underlying the suppression of AGEs-mediated inflammatory signaling, we assessed the influence of *Centella asiatica* on the phosphorylation of pivotal proteins within the AKT/MAPK signalling pathway, a regulator of the pro-inflammatory response in macrophages. The results indicated that *Centella asiatica* effectively attenuates the AGEs-induced activation of the AKT/MAPK pathway (*p* < 0.05, Figures 4J–M). Collectively, these outcomes have suggested that *Centella asiatica* intervention substantially diminishes the upregulation of inflammatory cytokines induced by AGEs, potentially due to the inhibition of the NF-κB pathway mediated by the AKT/AMPK signaling cascade.

**FIGURE 5:**
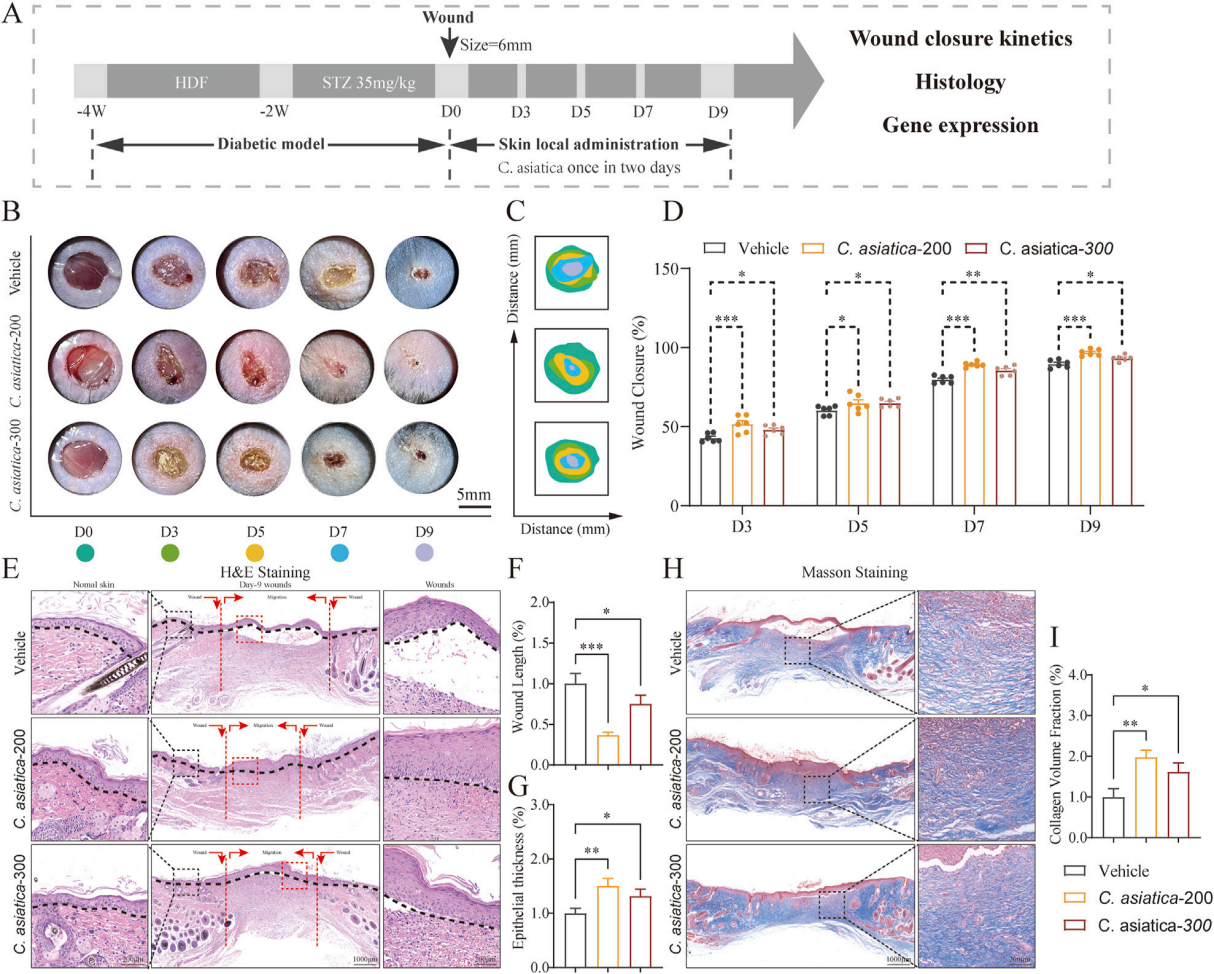
Evaluation of the wound healing process following *Centella asiatica* intervention *in vivo*. **(A)** Workflow for evaluating skin wound healing. **(B,C)** Representative images and mode patterns showing the process of wound closure in the Vehicle, *Centella asiatica*-200 μg/cm^2^, and *Centella asiatica*-300 μg/cm^2^ groups at days 0, 3, 5, 7, and 9 after the operation, treated with different drug concentrations. **(D)** Quantification of wound closure rates by using ImageJ software and was calculated by the percentages of wound closure compared to day 0 woundsize, conducted separate comparisons between the *Centella asiatica*-200 μg/cm^2^ group and *Centella asiatica*-300 μg/cm^2^ group against the Vehicle group, with better efficacy observed at *Centella asiatica*-200 μg/cm^2^, n = 6. **(E–G)** Quantitative evaluation of the gap widthof neo-epithelium and the degree of re-epithelization indicated by horizontal black lines, n = 3 (scale bar: 1,000 μm). **(H,I)** Cutaneous wound sections were subjected to masson’s trichrome staining, n = 3 (scale bar: 1,000 μm). The results were expressed as mean ± SD. *p < 0.05, **p < 0.01, ***p < 0.001; ns, not significant.

### Evaluation of the wound healing process following *Centella asiatica* intervention *in vivo*

The methodology for assessing the healing progression of diabetic wounds was depicted (Figure 5A). Both digital imaging and model photography were employed to illustrate the stages of wound healing in diabetic murine models after topical application of different concentrations of *Centella asiatica* on days 0,3,5,7 and 9 (Figures 5B,C). A comparative analysis of wound closure rates indicated that *Centella asiatica* significantly enhanced wound healing efficiency in diabetic wounds compared to the Vehicle group (*p* < 0.05, Figure 5D). Further quantitative evaluations of wound dimensions and the positioning of horizontal black lines in H&E-stained images highlighted that *Centella asiatica*’s influence on wound recovery and re-epithelialization markedly outperformed that of the Vehicle, with both dosages demonstrating therapeutic benefits, and optimal efficacy at 200 μg/cm^2^ (*p* < 0.05, Figures 5 E–G). Additionally, Masson’s trichrome staining was utilized to measure collagen deposition, revealing that treatment with *Centella asiatica* significantly promoted collagen synthesis compared to the Vehicle group (*p* < 0.05, Figures 5H, I). These findings suggest that *Centella asiatica* effectively accelerates the healing of diabetic wounds.

### *Centella asiatica* intervention enhanced proliferation and angiogenesis *in vivo*

**FIGURE 6:**
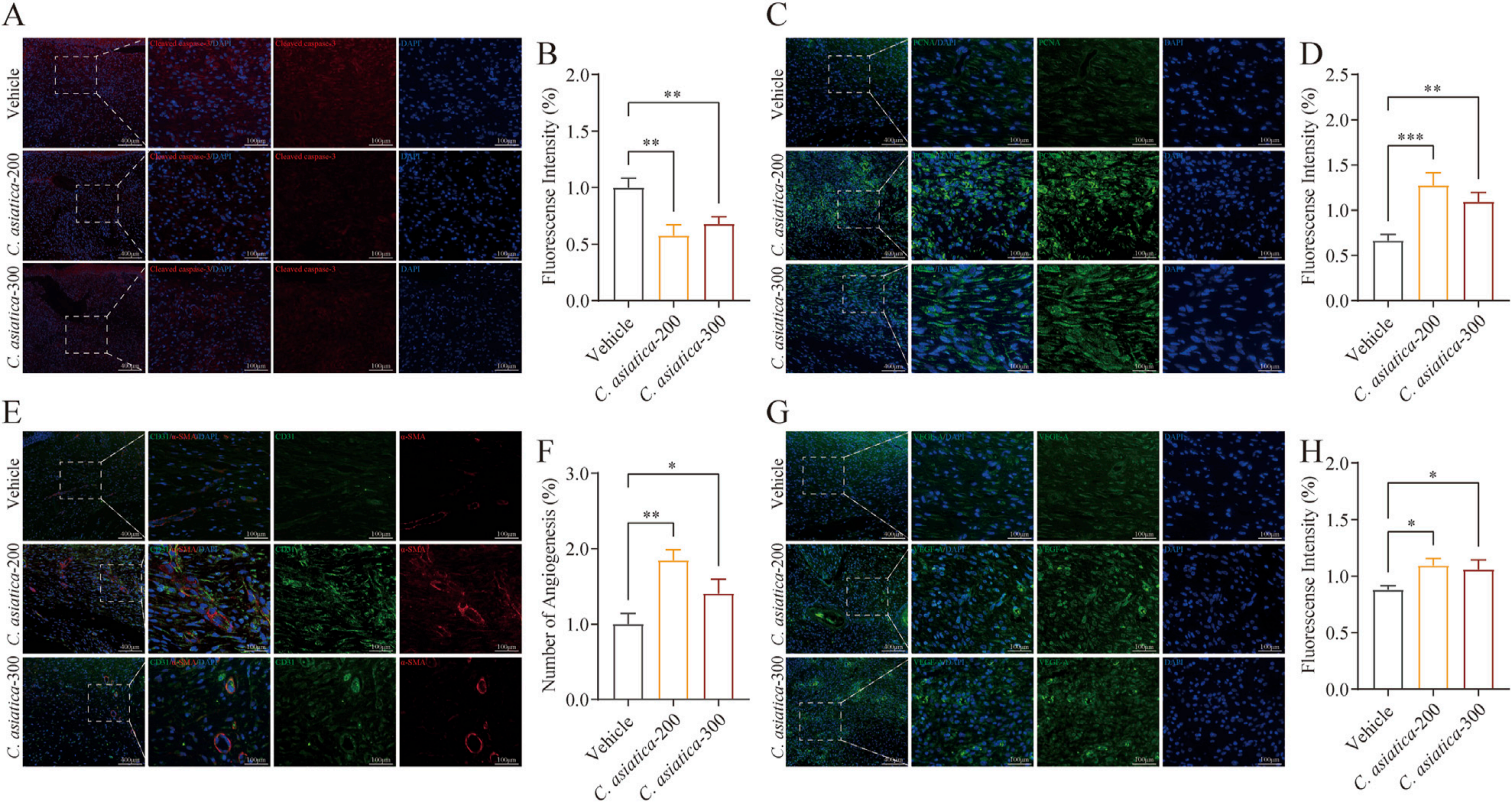
*Centella asiatica* promotes granulation tissue growth activity *in vivo*. **(A,B)** Representative immunofluorescence images and analyses showing cellular apoptosis (Red) in skin wounds on day 9 after intervention, n = 3 (scale bar: 100 μm). **(C,D)** Representative immunofluorescence images and analyses showing cellular proliferation (Green) in skin wounds on day 9 after intervention. The cell nucleus was stained with DAPI (Blue), n = 3 (scale bar: 100 μm). **(E,F)** Images and quantification of immunofluorescence staining for CD31 (Green) labelled with Alexa Fluor 488 and α-SMA (Red) labelled with Alexa Fluor 555 to reflect the degree of neovascularization, n = 3 (scale bar: 100 μm). **(G,H)** Representative immunofluorescence images and analyses for VEGF-A (Green) in skin wounds on day 9 after intervention, n = 3 (scale bar: 100 μm). The results were expressed as mean ± SD. *p < 0.05, **p < 0.01, ***p < 0.001; ns, not significant.

Building on the results that *Centella asiatica* could enhance the healing of diabetic wounds, we examined its impact on apoptosis and granulation tissue growth in diabetic wound healing. Immunofluorescence analysis indicated that treatment with *Centella asiatica* remarkably reduced cellular apoptosis at the edges of diabetic wounds (*p* < 0.05, Figures 6A,B). Proliferation assays further demonstrated that exposure to *Centella asiatica* significantly enhanced cell proliferation, underlining its potential role as a therapeutic agent for promoting wound healing in diabetic patients (*p* < 0.05, Figures 6C,D). To enhance our understanding of the effects of *Centella asiatica* on angiogenesis, we utilized dual staining techniques for CD31 and α-SMA, which serve as markers for endothelial and smooth muscle cells, respectively, to assess angiogenic activity. Results from imaging and statistical analyses revealed that *Centella asiatica* treatment facilitated increased angiogenesis, indicating its potential in enhancing vascular growth and tissue repair (*p* < 0.05, Figures 6E,F). Additionally, we evaluated the secretion of VEGF-A following *Centella asiatica* treatment and observed a significant increase in its levels (*p* < 0.05, Figures 6G,H). These findings have suggested that *Centella asiatica* accelerated diabetic wound healing by enhancing granulation tissue growth.

## Discussion

Wound healing in DFU encompasses a complex array of biological processes, including inflammation, oxidative stress, collagen synthesis, cellular migration, and proliferation (Mohsin et al., 2024). The inflammatory response is integral to preserving normal tissue function (Theocharidis et al., 2020). However, within the pathological milieu of diabetes, macrophage function is altered, and the conversion from the pro-inflammatory M1 phenotype to the anti-inflammatory M2 phenotype, a critical aspect of typical healing, is impeded (Li Y. et al., 2022; Lin et al., 2022). Consequently, inflammatory phases are extended, marked by the overproduction of cytokines such as TNF-α, IL-1β, and IL-6, exacerbating tissue damage and impeding healing responses in diabetic wounds. Thus, effectively modulating inflammation is crucial for diabetic wound repair. *Centella asiatica*, distinguished by its potent anti-inflammatory and antioxidant characteristics, has garnered considerable attention in treating various disorders, with proposed mechanisms that include anti-inflammatory activity, antioxidant effects, and modulation of glucose and lipid metabolism (Gray et al., 2016; Hussin et al., 2009; Lee et al., 2020; Qiu et al., 2022). Yet, its function in diabetic wound management remains to be fully clarified. Investigating the bioactive components and mechanisms of *Centella asiatica* in DFU treatment is, therefore, of paramount importance. Through the integration of systemic pharmacology with both *in vivo* and *in vitro* models, we elucidate the active components, potential targets, and associated signaling pathways that mediate the therapeutic effects of *Centella asiatica* on DFU.

In our work, Cynarine, Quercetin, and Kaempferol are identified as the main bioactive components of *Centella asiatica* for the treatment of DFU *via* systematic pharmacology combined with existing literature reports (Alqahtani et al., 2015). These compounds have demonstrated anti-inflammatory and antioxidant activities in recent research. Cynarine, specifically, has been shown to suppress the inflammatory pathways mediated by NF-κB and mitogen-activated protein kinases, and regulate the mRNA expression of key proteins in antioxidant defense such as Nrf2, Gpx, and SOD1 (Acquaviva et al., 2023; Kim et al., 2022). Similarly, Quercetin has been observed to attenuate inflammation (IL-1β, IL-6, and TNF-α) and oxidative stress in diabetic mouse models, such as db/db mice and STZ-induced diabetic mice (Yang et al., 2019). Additionally, related studies have also confirmed that the Kaempferol has a similar effect (Bian et al., 2022; Devi et al., 2015). Our pharmacological analysis indicates that the matrix metalloproteinase (MMP) family and estrogen receptors are extensively represented among the common targets of Centella asiatica-DFU. Notably, the signaling pathways associated with the MMP family and estrogen receptors are implicated in inflammatory processes, and members of the MMP family are significantly associated with the prognosis of DFU (Adachi et al., 2022; Li N. et al., 2022; Rui et al., 2024; Yao et al., 2014).

Abnormal inflammatory signaling is closely associated with impaired healing of diabetic wounds (Monaghan et al., 2023). Our work’s GO enrichment analysis suggests that *Centella asiatica* may modulate inflammation and oxidative stress, potentially playing a therapeutic role in diabetic wound management by modulating the inflammatory response. Further insights into the mechanism were gained through Reactome pathways enrichment analysis, which identified relevant signaling pathways and targets, including extra-nuclear estrogen signaling, PI3K/AKT signaling, tyrosine kinases, collagen degradation, activation of matrix metalloproteinases, and growth factor receptors signaling. Notably, extra-nuclear estrogen signaling, PI3K/AKT signaling and activation of matrix metalloproteinases are key regulators of immune responses (Adachi et al., 2022; Feng et al., 2023; Li N. et al., 2022; Yao et al., 2014). Thus, we posit that the bioactive components of *Centella asiatica* may contribute to diabetic wound healing by modulating the inflammatory response.

To further elucidate the anti-inflammatory properties of *Centella asiatica* in diabetic wounds, we employed RAW 264.7 murine macrophage cells to assess its effects *in vitro*. NO overproduction is detrimental in macrophage-mediated inflammatory responses (Wang et al., 2020). Our findings indicate that *Centella asiatica* markedly suppressed AGEs-triggered NO overproduction. Moreover, iNOS considerably mediates AGEs-induced NO secretion in macrophages, an action regulated by the pivotal nuclear transcription factor NF-κB, which in turn initiates transcription of pro-inflammatory mediators such as TNF-α, IL-6, and IL-1β (Chang et al., 2004; Harb et al., 2023). Our results show that *Centella asiatica* intervention attenuated NF-κB expression in a diabetic milieu, significantly ameliorating the mRNA expression levels of the pro-inflammatory cytokine genes IL-1β, IL-6, and TNF-α, while concurrently upregulating the expression of anti-inflammatory cytokines IL-4 and IL-10. Additionally, AGEs instigate macrophage inflammation by activating the downstream AKT/MAPK signaling pathway (Cui et al., 2022; Qin et al., 2013). Notably, an integral mechanism of natural products’ anti-inflammatory action involves AKT/MAPK inhibition (Cui et al., 2022; Hu et al., 2020; Tang et al., 2022; Zl et al., 2024). Hence, we explored the potential mechanistic relationship between *Centella asiatica* -mediated anti-inflammation and the AKT/MAPK pathway. The experimental outcomes imply that *Centella asiatica* substantially diminishes AKT/MAPK phosphorylation in RAW 264.7 cells under pathological conditions. Furthermore, in a subsequent *in vivo* efficacy evaluation, we substantiate the effectiveness of the *Centella asiatica* intervention in promoting diabetic wound healing. Our findings indicate that the local topical application of *Centella asiatica* significantly enhances the rate of wound healing, with optimal efficacy observed at a concentration of 200 μg/cm^2^. Through the assessment of pertinent indicators associated with wound granulation tissue regeneration, we establish the overall stimulatory effect of *Centella asiatica* on the regeneration of wound granulation tissue.

Collectively, our study demonstrated that the topical application of *Centella asiatica* significantly reduces AGEs-related inflammation at the cellular level and enhances the healing of diabetic wounds. These findings underscore the potential of *Centella asiatica* as a viable candidate for the treatment of DFU. However, its therapeutic potential is limited by poor aqueous solubility and low permeability, posing challenges for clinical application. These limitations necessitate the integration of additional materials or delivery systems to enable effective use in both *in vivo* and *in vitro* studies. *Centella asiatica* has been incorporated into various types of bioscaffolds to enhance its bioavailability, addressing the physicochemical limitations of Centella asiaticaand improving its efficacy in wound healing applications. By incorporating Centella asiaticainto 3D-designed bioscaffolds, including hydrogels, microneedle arrays, and nanofibrous mats, it exhibits synergistic capabilities with other biomaterials, creating possibilities for more effective treatments. While preclinical studies are promising, clinical trials are crucial to evaluate the efficacy and safety of asiaticoside-based bioscaffolds in patients with DFU. Overall, *Centella asiatica* shows promise as a versatile therapeutic agent, requiring further research to enhance its bioavailability and clinical potential. Despite the insights gained from our mechanistic and preclinical evaluations, this study has several limitations. Firstly, while we demonstrated the efficacy of Centella asiatica-derived bioactive metabolitess in modulating macrophage polarization and inflammatory pathways, the long-term safety and pharmacokinetic profiles of these compounds in the context of DFU healing remain underexplored. Secondly, the translational potential of our findings is constrained by the lack of clinical data. These limitations emphasize the necessity for expanded preclinical and clinical investigations to advance the therapeutic application of *Centella asiatica* in DFU management.

## Conclusion

DFU represent complex conditions influenced by a multitude of factors, including immune dysregulation, chronic inflammation, and metabolic disorders. *Centella asiatica* has emerged as a promising therapeutic agent due to its potent biological activities, which include a range of wound-healing properties. Our investigation established a significant correlation between the active constituents of *Centella asiatica* and macrophage immune modulation *in vitro*. Furthermore, we demonstrated its efficacy in promoting diabetic wound healing through experiments *in vivo*. This study provides initial validation of the bioactive compounds present in *Centella asiatica* and offers a preliminary exploration of its anti-inflammatory activities and underlying mechanisms. These findings contribute to the development of more effective and safer treatment options, thereby enhancing future therapeutic strategies for the management of DFU.

## Data Availability

The original contributions presented in the study are included in the article/Supplementary Material, further inquiries can be directed to the corresponding authors.

## Abbreviations

AGEs, advanced glycation end products; *Centella asiatica*, *centella asiatica*; DFU, diabetic foot ulcer; GO, gene ontology; NO, nitric oxide; ROS, reactive oxygen species; STZ, streptozotocin; TCM, traditional chinese medicine; HFD, high-fat diet; iNOS, inducible nitric oxide synthase; Raw264.7, mouse mononuclear macrophage cells; MMP, matrix metalloproteinase; BP, biological process; CC, cellular component; MF, molecular function.
